# Radical resection of trigeminal schwannoma at the cerebellopontine angle with support of the digital robotic exoscope Synaptive Modus V system: A case report and literature review

**DOI:** 10.1097/MD.0000000000033492

**Published:** 2022-04-07

**Authors:** Tan-Si Chu, Tan-Huy Chu, Tri-Dung Huynh, Van-Dinh Phan, Bao Ngoc Dang, Quoc Dat Tran

**Affiliations:** a Department of Neurosurgery, Tam Anh Hospital, Ho Chi Minh City, Vietnam; b Department of Hematology, Pham Ngoc Thach University of Medicine, Ho Chi Minh City, Vietnam.

**Keywords:** digital robotic exoscope Synaptive Modus V system, trigeminal schwannoma, tumor of the cerebellopontine angle

## Abstract

We report on a 67-year-old woman who experienced drowsy mental status, facial numbness, and hearing loss on the right side; the symptom gradually worsened over the last 4 years. Brain magnetic resonance imaging revealed a 4.8 × 1.8 × 2.6 cm lesion located in the right cerebellopontine angle.

**Interventions:**

She underwent surgery with the retrosigmoid suboccipital approach and support from the digital robotic exoscope Synaptive Modus V system. To the best of our knowledge, this is the first reported case that used the robotic exoscope system in Vietnam, and also in Asia.

**Diagnosis:**

We performed radical resection of the tumor, the surgery position and the pathology result concluded the diagnosis was trigeminal schwannoma.

**Outcomes:**

After 30 months of follow-up, she fully recovered and the magnetic resonance imaging showed radical resection of the tumor.

**Lessons:**

The aim of this study is to share our experience with the robotic exoscope system, which can improve optical field and image resolution, hence creating an opportunity for surgery that otherwise is impossible. The application of this robotic exoscope system is a breakthrough in neurosurgery in developing countries, such as Vietnam.

## 1. Introduction

The cerebellopontine angle (CPA) is a triangular space in the posterior cranial fossa. It is an important landmark of anatomy and clinical, which is occupied by the CPA cistern, including the cranial nerves V, VI, VII, and VIII along with the cerebellar artery. Tumors of the CPA account for around 5% of intracranial neoplasm.^[1]^ The most frequent type of tumors are vestibular schwannomas, followed by meningiomas and epidermoid tumors; much more rare tumor of CPA are schwannomas of other cranial nerves. The treatment options for tumors of CPA include observation, radiation therapy, and surgery.^[1,2]^ Surgery can offer a definitive solution, however, due to the critical anatomical position of CPA, the surgery option was often put into hesitation.^[1]^

The introduction of the surgical microscope greatly impacted the neurosurgery field. The current surgical microscopes have gone through major updates, but still, it has some limitations. Firstly, the binocular lenses are attached to the microscope, which requires handling thus affecting the comfort of surgeons and prolonging the surgery duration. Secondly, illumination during surgery in-depth has some limitations that can affect the surgical outcome. The Synaptive Modus V system is fully automated, it provides a hands-free robotic movement of the camera and optical focal depth control via a tracked surgical instrument, with an enhanced LED light sources surrounding the camera.^[3]^ Thus, the robotic exoscope system can resolve the surgical microscope problems, and bring better outcomes for the patient. Here, we report an immensely challenging case of tumor in the right cerebellopontine angle, which was successfully treated by surgery with support of the digital robotic exoscope Synaptive Modus V system

## 2. Material and method

The Modus V (Synaptive, Cananda) is a fully automated 2-dimensional exoscope, with tracked surgical instrument that provides hands-free robotic movement of the camera and optical focal depth control. The main surgeon had an experience of more than 20 hours of practising with the system in the lab setting before the surgery. For case series data, we searched PubMed using the keywords “Trigeminal Schwannoma” and “Microsurgery”; In total, 355 reported cases were reviewed, we extracted relevant data and created Table 1.

**Table 1:** Trigeminal schwannoma reported cases comparison. In total, 355 reported cases with conventional surgical treatment were analyzed.

Author	Year	Total case†	Radical removal (%)	Mortality (%)	Morbidity (%)*
Yoshida and Kawase^[4]^	1999	27	20 (74)	0	74
Goel et al^[5]^	2003	73	51 (70)	3	72.6
Pamir et al^[6]^	2007	18	17 (94)	0	28
Wanibuchi et al^[7]^	2012	105	86 (82)	0	84
Chen et al^[8]^	2014	55	52 (95)	0	72
Samii et al^[9]^	2014	20	15 (75)	0	85
Neves et al^[10]^	2019	14	10 (71)	0	57
Li et al^[11]^	2021	43	39 (91)	2.3	82.8

* Morbidity data presented in prevalence. The data included all the tumor classifications based on their location.

† In total, 355 reported cases were analyzed.

## 3. Case presentation

We report on a 67-year-old woman who experienced vertigo, headache, facial numbness, and hearing loss on the right side. Over the last 4 years, she went to many hospitals in Vietnam, with the diagnosis of tumor in the right CPA, however, due to the critical position of tumor, the microsurgery option was postponed, and the patient was put on observation. She was treated only to reduce her symptom.

**Figure 1.**
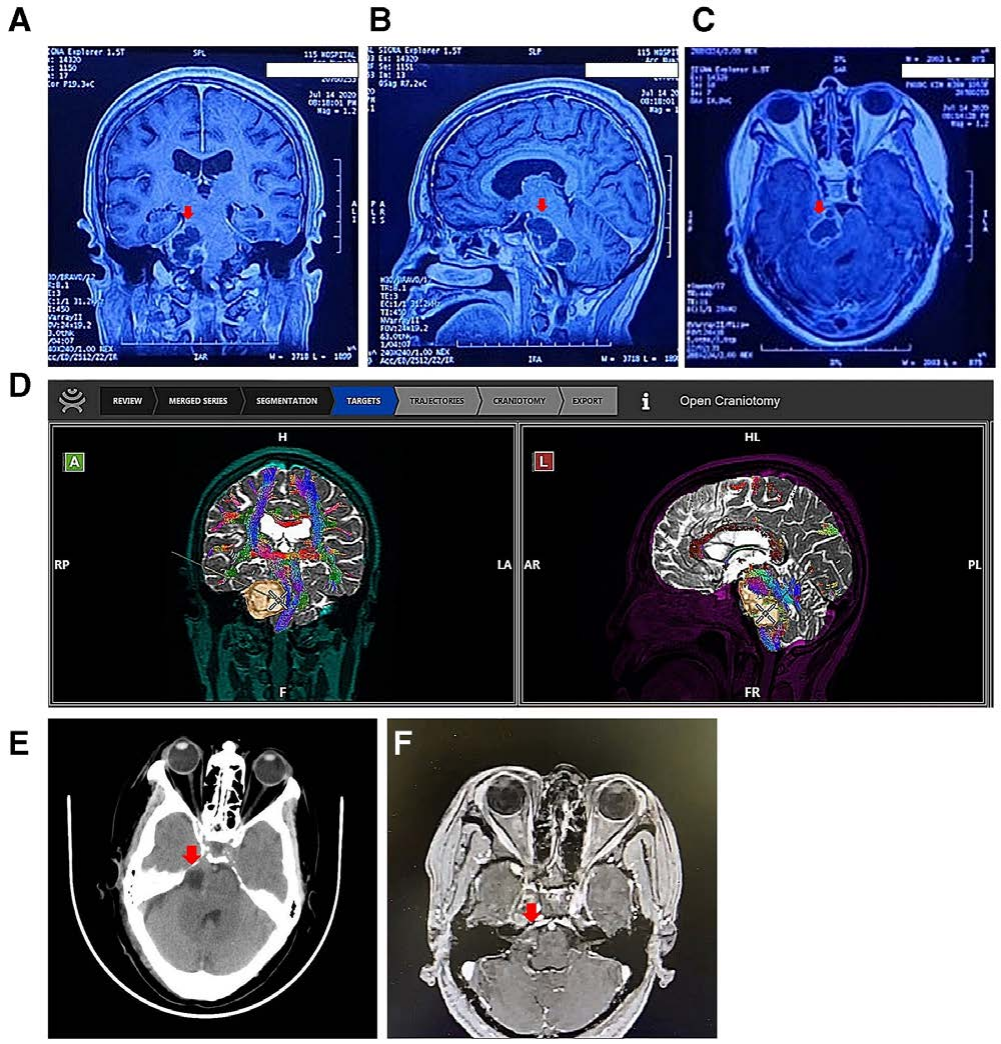
The tumor at CPA in MRI before the operation. A 4.8 × 1.8 × 2.6 cm uniform density tumor at the right CPA in T1-weighted MRI, in all 3 planes: (A) axial, (B) sagittal, and (C) coronal view, (D) and with DTI view of the tumor. Red arrow point at the tumor position. (E) The coronal view, 3 months after the operation. (F) The coronal view, 24 months after the operation. Red arrow points at the tumor position. CPA = cerebellopontine angle, DTI = diffusion tensor imaging, MRI = magnetic resonance imaging.

**Figure 2.**
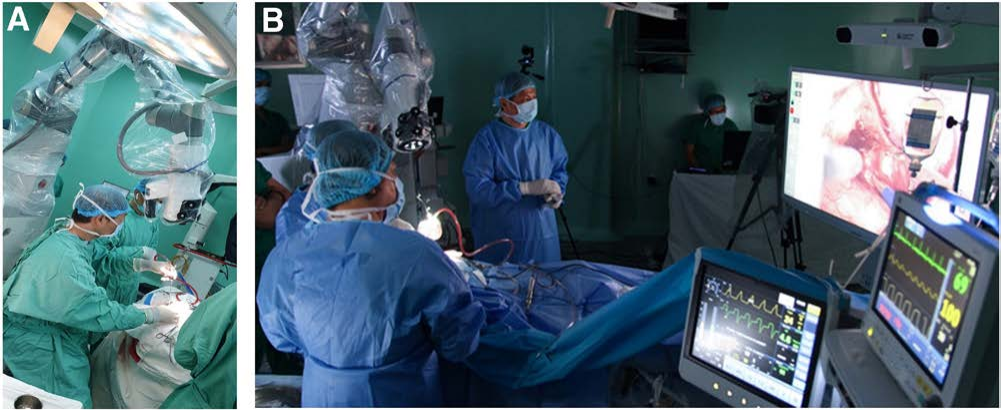
The digital robotic exoscope Synaptive Modus V system support our main surgeon in the operation. Representative picture, (A) the Modus V was set up above our main surgeon with a tracked surgical instrument to provide hands-free movement of the camera. (B) Combine with a digital medical-grade monitor, Modus V system can predict lighting and camera conditions to provide an optimal surgical field.

On the physical examination, the blood pressure, pulse, temperature, and respiration rate were 120/70 mm Hg, 76/min, 36°C, and 20/minutes, respectively, the patient’s condition overall is poor. Neurological examination revealed Glasgow Coma Scale score of 15 points, drowsy mental status, with peripheral cranial nerves V, VII, and VIII palsy on the right side. In detail, the examination of the cranial nerve V showed decreased sensation in the right half of the face according to the dominant region of V2. The patient had moderate hearing loss, with Weber test combined with Rinne test results showing sensorineural hearing loss on the right side. The cranial nerve VII on the right has mild paralysis. The cranial nerves IX, X, XI, XII have no abnormal signs. Brain magnetic resonance imaging (MRI) revealed a 4.8 × 1.8 × 2.6 cm uniform density lesion located in the right cerebellopontine angle, in association with severe compression of the brainstem and fourth ventricle (Fig. 1A–C). The diffusion tensor imaging (DTI) images by MRI system provided the anatomical information about the lesion, then the DTI images were used for practising with the robotic exoscope system, and can be saved for later reference (Fig. 1D). On Day 3 after hospital admission, the patient underwent surgery with the retrosigmoid sub-occipital approach with support from the digital robotic exoscope Synaptive Modus V system (Fig. 2A and B). We retracted the cerebellar to the left side and approached the tumor. The tumor is well-defined, encapsulated, and is an extra-axial tumor. We opened the capsule and removed the tumor part by part in the capsule. Next, we dissected the encapsulated tumor, release the tension of the tumor from the pons and the cranial nerves. Eventually, we stopped the bleeding, closed the dura mater, and put the skull cap back on. To the best of our knowledge, this is one of the first cases using the support of the Synaptive Modus V system in Vietnam, and also in Asia, which is documented by the ASIA book of records.^[12]^ We performed radical resection of the tumor in a 4-hour operation, with minimal bleeding and all of the important structures were preserved. The pathology result of the tumor sample was schwannoma, and both the MRI images and the surgery position is on cranial nerve V, which concludes the diagnosis of trigeminal schwannoma. The patient’s condition improved at 24 hours after the operation (Day 4), and was discharged at 96 hours, which is Day 7 after hospital admission. After 30 months of follow-up, she fully recovered. In detail, the patient feels very slight numbness in the right half of the face according to the V2-dominant region and still improving, she can hear whispers and snapping fingers, with Weber test combined with Rinne test results showing equal hearing in both ears, thus a manifestation of full recovery of hearing ability. The cranial nerves VII, IX, X, XI, XII have no abnormal signs. The MRI at 3 and 24 months of follow up showed complete removal of the tumor without any recurrence (Fig. 1E and F**).**

## 4. Discussion

Trigeminal schwannomas (TS) is a rare tumor that accounts for less than 0.1% of all intracranial tumor.^[1]^ Patients with TS usually present with facial numbness and neuralgia due to trigeminal nerve dysfunction, and/or the paralysis of other cranial nerves because of tumor compression; and/or other symptoms due to increased intracranial pressure.^[10,11]^ Although stereotactic radiosurgery has been developed over the last few decades, surgical resection is still regarded as the gold standard treatment.^[1,11]^ It needs to be remembered that any surgical approach is quite a challenge in treating the CPA tumors, as it involves immensely complex regional anatomy, with vital structures present in the surgical field; thus allowing a very narrow surgical corridor to operate^[1,9]^

Four years ago, our patient initially presented with vertigo and headache. She went to many hospitals in Vietnam and was diagnosed with a tumor in the right CPA. Nevertheless, because of the critical anatomical position, the observation approach is chosen with the assumption that the tumor will stop growing or undergo spontaneous regression. However, other studies reported that the majority of the CPA tumors exhibit further growth^[1,2]^; similar to previous reports, in this case, the tumor continued to grow, and the patient’s symptoms gradually worsened over the last 4 years. She was admitted to our hospital with poor overall condition, drowsy mental status, and cranial nerves palsy. With the tumor that have a maximal diameter of 4.8 cm, thus the stereotactic radiosurgery is not recommended,^[2]^ and the surgery approach was chosen, with the ultimate goal is not only a removal of the big tumor at CPA but also functional preservation of all cranial nerves and recovery of quality of life.^[1,10,11]^

The strengths of exoscopes are the wide view and deep focus with good illumination that can reduce the need for repositioning and refocusing during the surgery, hence better visual which is critical for surgery. Published data on the VITOM-3D, ORBEYE, and Modus V exoscope systems reported comparable results with the conventional surgical microscope with better image quality and illumination in surgeries.^[3,13,14]^ In this case, we chose the retrosigmoid sub-occipital approach and the supprot from Modus V system, which can offer a good visual of the whole CPA and increase safety during tumor dissection from brainstem and lower cranial nerves. According to Li et al,^[11]^ reported on 43 cases with trigeminal schwannoma received conventional surgical treatment, the postoperative rate of facial numbness remain at 82.8% of patients, other publications also reported similar data with associated morbidity was high at around 57% to 85%^[4–10]^ (Table 1). Mainly due to only partial removal of the tumor, and TS arise from trigeminal nerve fiber which is hard to be recognized and protected during the operation. With the help of this robotic exoscope system, we can clearly observed the surgical field, radical remove the tumor and preserve all the nerves, resulting in the patient’s complete recovery. To the best of our knowledge, this is the first reported case that used the robotic exoscope system in Vietnam, and also in Asia.^[12]^ The aim of this study is to share our positive initial experiences with the Modus V system, and we plan on reporting our clinical trial with the system.

In conclusion, the digital robotic exoscope Synaptive Modus V system is a safe and helpful tool to perform intracranial tumor resection. Especially, the artificial intelligence in the system provides hands-free movement, which gives a time-saving factor and comfortable for surgeons. The optical field and the image resolution are better than that of a surgical microscope. These factors help to reduce the length of the operation, minimize the risk of complications, hence creating an opportunity for surgery that otherwise is impossible, and bringing better outcomes for the patient. The application of this robotic exoscope system is a breakthrough in neurosurgery in developing countries, such as Vietnam.

## Acknowledgments

We would like to say our special thanks to Quynh Nguyen Xuan Thuy, Department of Infectious Diseases, Children’s Hospital no.2, Ho Chi Minh City, Vietnam; and Tan-Son Chu, Department of Medicine, Nguyen Tat Thanh University, Ho Chi Minh City, Vietnam; for their contribution to English correction, and literature review. We would like to say our special thanks to the faculty of People’s Hospital 115, Ho Chi Minh City, Vietnam; and the Department of Neurosurgery, Pham Ngoc Thach University of Medicine, Ho Chi Minh City, Vietnam; for their contribution to medical support.

## Author contributions

**Conceptualization:** Tan-Si Chu.

**Data curation:** Tan-Si Chu, Tri-Dung Huynh, Van-Dinh Phan, Bao Ngoc Dang, Quoc Dat Tran.

**Funding acquisition:** Tan-Si Chu.

**Investigation:** Tan-Si Chu, Van-Dinh Phan, Bao Ngoc Dang, Quoc Dat Tran.

**Methodology:** Tan-Si Chu, Tri-Dung Huynh.

**Project administration:** Tan-Si Chu.

**Supervision:** Tan-Huy Chu.

**Validation:** Tan-Huy Chu.

**Visualization:** Tan-Huy Chu.

**Writing – original draft:** Tan-Huy Chu.

**Writing – review & editing:** Tan-Huy Chu.
